# Supplementary material for: Polygenic scores for autism are associated with neurite density in adults and children from the general population
Source: medRxiv. 2024 Apr 13:2024.04.10.24305539. Preprint. [Version 1] doi: 10.1101/2024.04.10.24305539 (PMC11030520; doi:10.1101/2024.04.10.24305539)
Supplement: Supplement 1 [file media-1.docx]

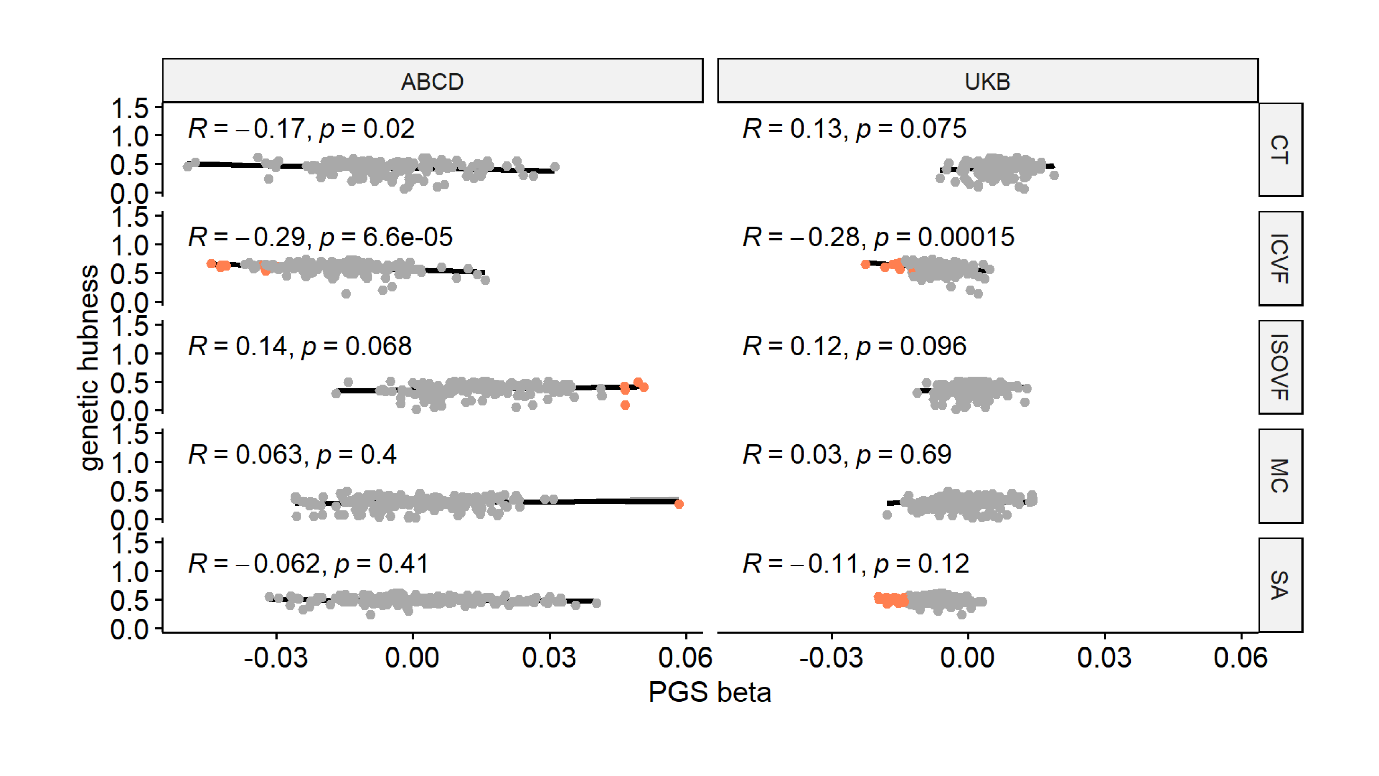


**Supplementary Figure 1. Enrichment Analysis:** *Correlation plot between genetic hubness and autism PGS association across MRI-derived phenotypes of interest in the ABCD and UK Biobank cohorts. Each point represents a region and regions where there is a significant association between the phenotype and autism PGS are coloured in red. R is the Pearson correlation coefficient. p is the p value before multiple testing correction.*


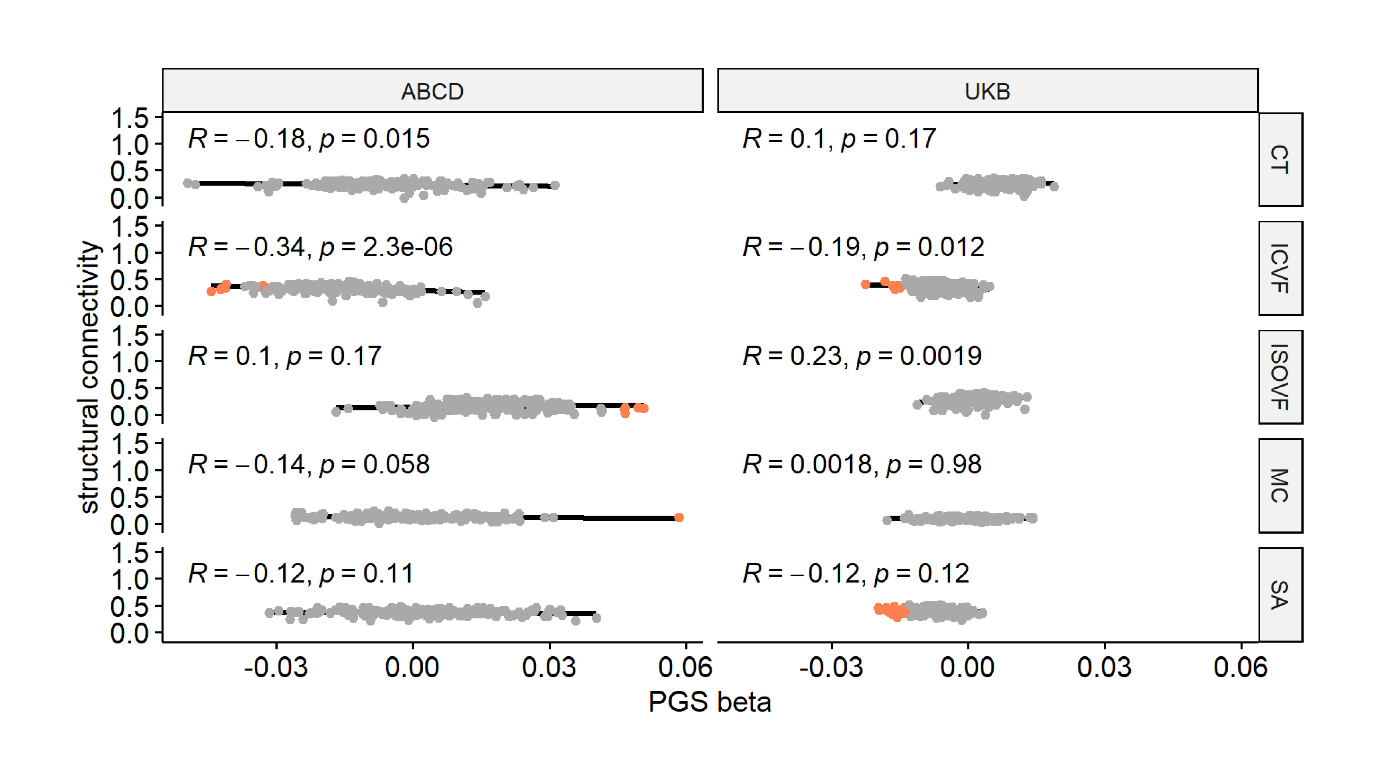


**Supplementary Figure 2. Enrichment Analysis:** *Correlation plot between structural connectivity and autism PGS association across MRI-derived phenotypes of interest in the ABCD and UK Biobank cohorts. Each point represents a region and regions where there is a significant association between the phenotype and autism PGS are coloured in red. R is the Pearson correlation coefficient. p is the p value before multiple testing correction.*
